# Machining behavior and phase transformation in WEDM of Ti₅₀Ni₄₉Co₁ shape memory alloy

**DOI:** 10.1038/s41598-026-53849-x

**Published:** 2026-06-02

**Authors:** Hargovind Soni, R Suresh Kumar, G. N. Kumaraswamy, M Madhusudan, C. Solaimuthu, Ritesh Verma, Nidhin A Raj, Priyaranjan Sharma

**Affiliations:** 1 Department of Mechanical Engineering, Galgotias University, Greater Noida, 203201, Uttar Pradesh India; 2 Department of Mechanical Engineering, BMS College of Engineering, Bengaluru, 560019, Karnataka India; 3 Department of Physics, Amrita School of Engineering, Amrita Vishwa Vidyapeetham, Bengaluru, 560035, Karnataka India; 4 Department of Mechanical Engineering, Dayananda Sagar College of Engineering, Bengaluru, 560111 Karnataka India; 5 Department of Mechanical Engineering, RV Institute of Technology and Management, Bengaluru, 560076 Karnataka India; 6 Department of Physics, Graphic Era Deemed to be University Dehradun, Dehradun, 248002 Uttrakhand India; 7 Department of Mechanical Engineering, Adi Shankara Institute of Engineering & Technology, Ernakulam, 683574 Kerala India; 8 Department of Mechanical Engineering, Manipal University Jaipur, Dehmi Kalan, Jaipur, 303007 Rajasthan India

**Keywords:** Recast layer formation, Material removal rate (MRR), Surface integrity, TiNiCo Shape Memory Alloy, Phase Transformation (DSC/XRD), X-ray diffraction (XRD), Engineering, Materials science

## Abstract

The study reported in this research article focuses on the machining behavior and phase transformation of Ti₅₀Ni₄₉Co₁ shape memory alloy during wire-electrical-discharge-machining (WEDM). Experiments were performed to determine the combined effect of the pulse-on time (Ton) and servo voltage (SV) under high-energy discharge conditions, using a Taguchi L_25_ orthogonal array. The results show that the pulse-on time significantly increases discharge energy, which reached a peak MRR of 8.43 mm^3^/min with Ton = 125 µs and SV = 20 V (Run order 21). However, it also increased the surface roughness (Ra) from 2.85 μm to 5.01 μm, producing deeper craters and hardened recast layers as well as re-solidified debris because of rapid melting and solidification. Major morphological changes were observed at high discharge energy, as confirmed by 3D surface profilometry. The servo voltage influenced the stability of the spark: higher voltages reduced discharge intensity, resulting in lower material removal rate but improved surface finish. XRD analysis revealed the formation of secondary phases, containing NiTi_2_, TiO_2_, and CuZn, was produced during machining, resulting from oxidation and transfer of the electrode material. DSC examination showed an increase in transformation peaks, a decrease in transformation enthalpy, and an insignificant change in transformation temperatures, indicating that the martensitic transformation in the recast layer was partially suppressed by compositional alterations and oxide formation. Nevertheless, no loss of shape-memory property was observed in the bulk material, although slight variations in transformation behavior were observed. Overall, the results support the importance of properly optimizing pulse-on time and servo voltage to achieve a balance among good machining performance, consistent surface quality, and good functional performance, particularly in precision biomedical, aerospace, and smart actuator applications.

## Introduction

Shape memory alloys (SMAs) are advanced functional materials that can recover their original shape upon heating or unloading due to a reversible martensitic–austenitic phase transformation^1^. TiNi-based alloys are among these materials and have attracted considerable attention due to their excellent properties, such as high strength, good corrosion resistance, and high biocompatibility^2^. The stability of transformation, mechanical strength, and fatigue resistance are further increased by the addition of alloying elements such as cobalt (Co), and thus TiNiCo SMAs are specifically used in biomedical implants, aerospace actuators, and smart structural elements^3^. Nevertheless, it is extremly difficult to machine TiNi-based SMAs using traditional machining methods because of their high work-hardening tendency and low thermal conductivity. Such properties contribute to rapid tool wear, inappropriate surface finish, and thermal damage in traditional cutting operations. Therefore, non-traditional machining methods, such as Wire Electrical Discharge Machining (WEDM), have consequently become useful for providing alternatives to machining hard-to-cut materials with high precision and low mechanical stress^4,5^.

WEDM is a machining process that uses a thermo-electric process where material is removed by controlled electrical discharges between a continuously moving wire electrode and the workpiece in a dielectric medium^6^. The process allows the cutting of complicated shapes and hard materials with extremely high dimensional accuracy. Though the material removal rate (MRR) and surface roughness (SR) have these benefits, the balance between the two is not easy due to the high sensitivity of the two variables to the parameters of the electrical discharge^7,8^. Other process parameters that define the WEDM process include the pulse-on time (Ton) and the servo voltage (SV) which are relevant to spark energy, discharge stability and effectiveness in removing the debris.

The pulse-on time influences the amount of time during which energy is emitted in each discharge and consequently directly affects the thermal load and the erosion rate. Conversely, the inter-electrode distance is controlled by the servo voltage and influences the intensity of discharge and the integrity of the surface^9^. Past research has shown that increasing pulse-on time can usually increase MRR, though it is likely to degrade the surface finish by over-melting and re-solidifying the material. Similarly, the servo voltage range has been shown to be important for discharge stability, crater development, and overall surface morphology during machining^10,11^.

The shape memory alloys based on NiTi have attracted significant research due to their unique functional properties, including the shape memory effect, superelasticity, and high corrosion resistance. All these features make them particularly useful for aerospace components, biomedical devices, and intelligent actuation systems. Recent advances in manufacturing technologies, particularly additive manufacturing, have enabled the production of complex NiTi-based parts with tailored microstructures and functional properties^12–16^. Nevertheless, ensuring consistent machining and maintaining normal functioning are the main concerns in the production of such alloys.

Although certain WEDM operations are highly advantageous for machining NiTi alloys, the thermoelectricity of the process may significantly influence surface integrity and microstructural properties. During machining, the high energy of the spark causes local melting, followed by rapid solidification, which may introduce microcracks, re-solidified globules, and recast layers. These are the surface characteristics that can adversely affect the mechanical and functional behavior of the machined parts. For example, Kulkarni et al.^17^ reported that pulse-on time and servo voltage increase the spark energy in WEDM of medical-grade NiTi alloys, leading to deep craters, recast thickening, and the formation of microcracks on the machined surface. Their SEM analysis shows a textured surface morphology, a product of clusters of molten droplets and debris, typical of thermal erosion during WEDM.

Similarly, other researchers have demonstrated that the surface integrity and machinability of NiTi-based alloys are highly affected by process parameters. Studies of NiTiHf shape memory alloys have shown that the greater the heat added to the machining process, the more material is melted and solidified, and therefore the greater the surface roughness and microstructural inhomogeneities^18,19^. Besides the surface morphology, machining thermal effects may also affect the phase transformation behavior of the shape memory alloys. Surface processing technologies such as EDM and laser machining may also add residual stress, compositional alterations, and microstructural defects which cause martensitic transformation temperatures and operational stability to change^20–22^.

This is particularly pertinent when critical control of transformation temperatures is necessary, e.g., in actuators, elasto-caloric systems, and biomedical implants^23^. Also, it can affect the transformation stability because recast layers and localized compositional variations can be produced during EDM machining, thereby negatively influencing the alloy’s cyclic functional behavior^20,22^.

The machining of TiNiCo shape memory alloy with WEDM thus presents a critical problem: high discharge energy can enhance material removal, but it also weakens surface integrity, encourages the growth of unwanted secondary phases, and inhibits martensitic transformation, thereby decreasing the functionality of the alloy. Even though TiNi-based SMAs are finding applications in biomedical, aerospace, and actuator applications, where both high productivity and constant functional behavior are needed, making this tradeoff in machining is not easy. Moreover, the available literature does not provide a closer relationship between surface topography and various phase transformations in WEDM-machined TiNi-based SMAs using the analysis of differential scanning calorimetry (DSC)-based transformation behavior, which evokes the necessity of a systematic study of the relationship between machining parameters and surface characteristics in parallel, as well as their influence on the occurrence of thermally driven phase changes.

The novel approach to defining a direct relationship between machining parameters, microstructural evolution, surface damage, and transformation instability in TiNiCo SMAs is based on an integrated experimental method that includes a Taguchi L_25_ orthogonal design, X-ray diffraction (XRD), differential scanning calorimetry (DSC), and three-dimensional surface profiling. The key goal is to establish the most effective pulse-on time/servo voltage trade-off, which allows attaining a sensible balance between machining efficiency, low surface degradation, and stable shape-memory behavior, and facilitates the stable use of TiNiCo shape-memory alloys in precision engineering systems.

## Materials and methods

### Material Characterization

**Fig. 1:**
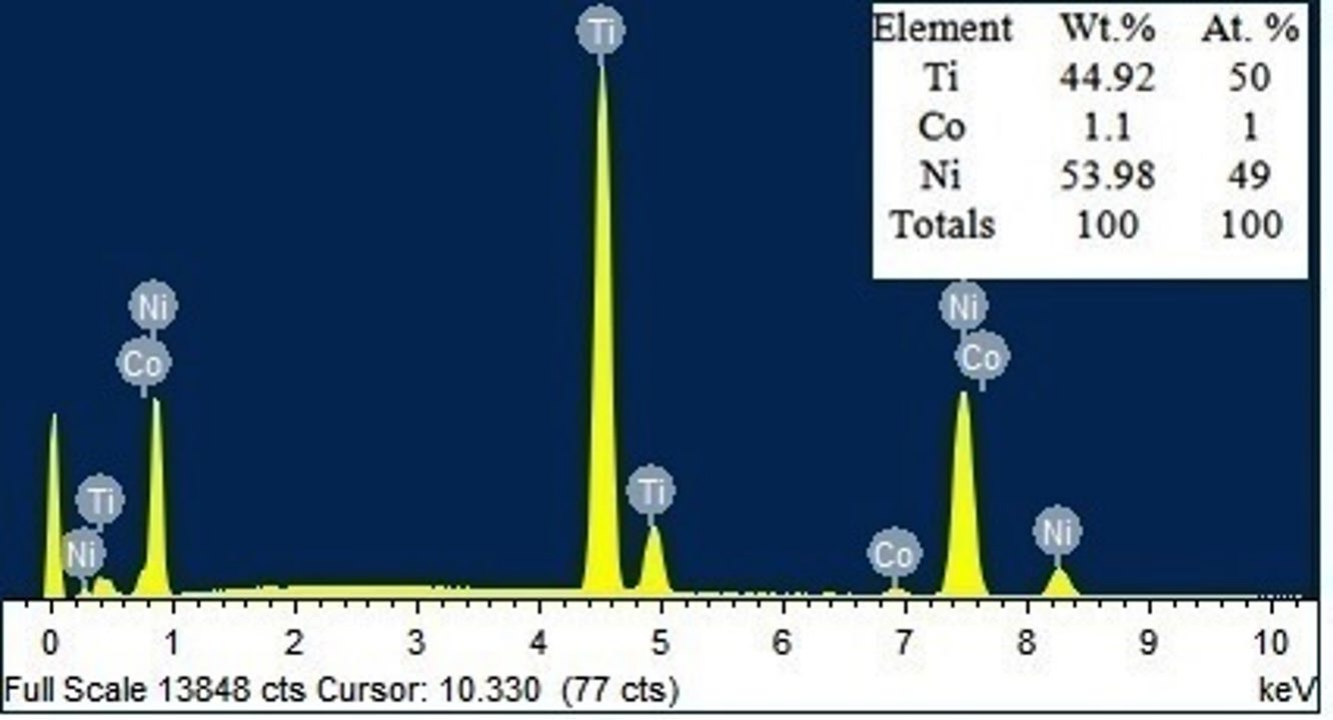
EDX analysis of as-cast Ti₅₀Ni₄₉Co₁ alloy.

Energy-dispersive spectroscopy (EDS) was performed to examine the phase structure and chemical composition of the developed SMA, as shown in Fig. 1, and the results revealed the compositional integrity of the Ti₅₀Ni₄₉Co₁ shape memory alloy (SMA)^3^. The alloy was analyzed by elemental analysis, which showed that it contained Ti, Ni, and Co. Cobalt was intentionally added to modify the phase stability and martensitic transformation properties of TiNi-based SMAs. The introduction of cobalt partially substitutes Ni lattice sites, changes transformation temperatures, and stabilizes the B2 austenite phase, thereby influencing microstructural formation during the thermal process. Such compositional modification is particularly relevant for the present study, as it can influence the thermo-electrical behavior of the alloy during WEDM process and affect the discharge properties, the formation of recast layers, and the overall surface integrity.

**Fig. 2:**
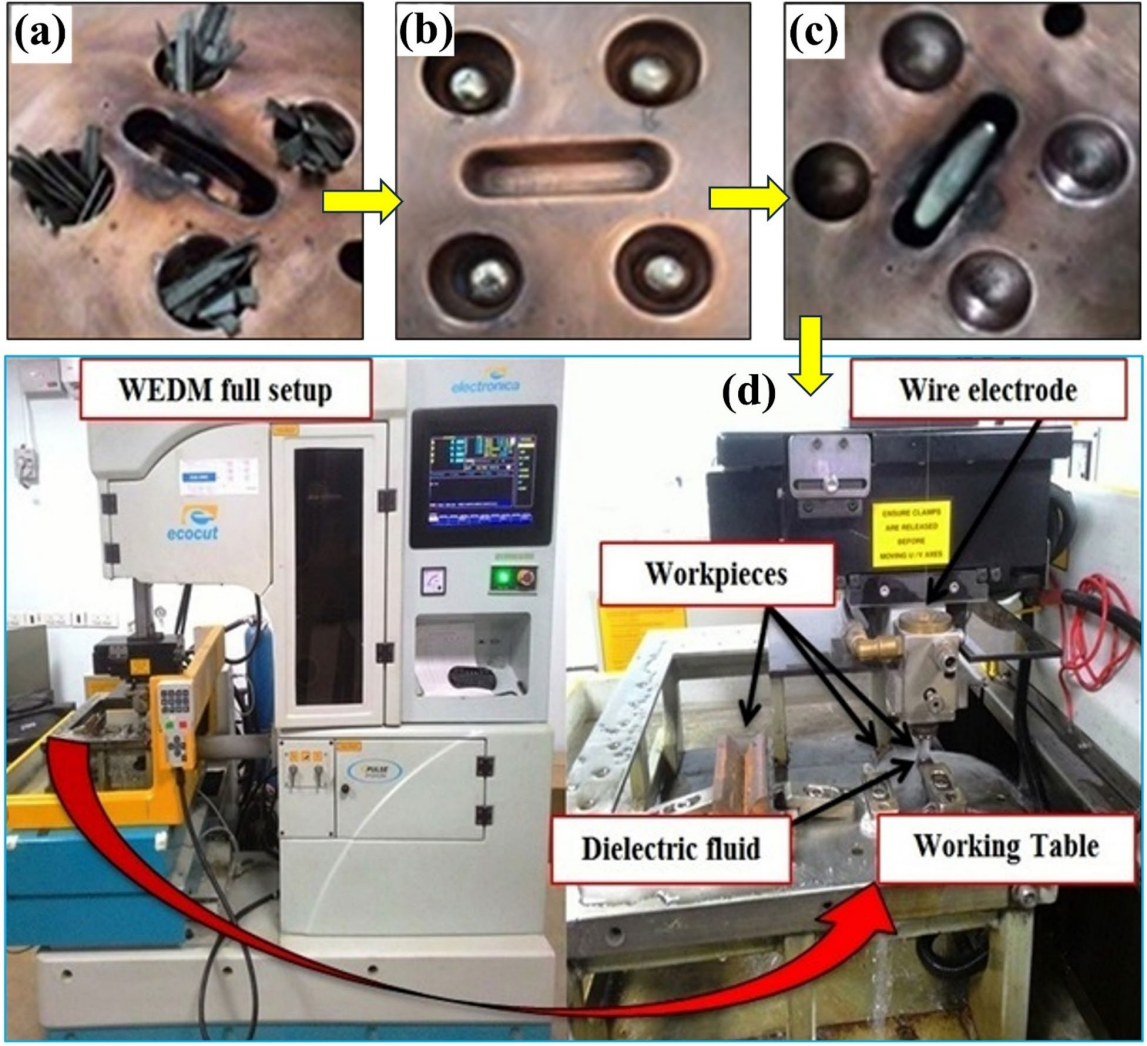
Experimental workflow: (**a**) vacuum arc melting of Ti, Ni, and Co ingots, (**b**) sectioned Ti₅₀Ni₄₉Co₁ specimen with dimensions of (10 × 10 × 5 mm), (**c**) final specimen prepared for WEDM machining experiments, and (**d**) WEDM experimental setup used for machining the Ti₅₀Ni₄₉Co₁ alloy.

Figure 2(a-c) shows the preparation procedure of the specimen. A vacuum arc-melting furnace was used to produce the Ti₅₀Ni₄₉Co₁ alloy, in which commercial Ti, Ni, and Co ingots were melted in a water-cooled copper crucible under a high-purity argon atmosphere to prevent oxidation. Melting was performed under vacuum (approximately 10^− 6^ mbar), and the vacuum was then replaced with argon to maintain an inert atmosphere. To achieve a homogeneous alloy, the alloy was melted many times. The hardened ingot was then cut into 10 mm × 10 mm × 5 mm samples for WEDM machining experiments in the as-cast state.

### WEDM experimental setup and parameters

Figure 2(d) shows the WEDM machining set-up in the present study. The experiments were done with an Electronica EcoCut Pulse 15 WEDM machine (Make: Electronica Machine Tools Ltd., India). The system incorporates necessary machining components, including the wire-feeding system, dielectric circulation system, and servo control system, to maintain the spark gap throughout machining.

According to earlier research, pulse-on time (Ton) and servo voltage (SV) were found to be the most sensitive WEDM control factors influencing both MRR and surface integrity^24^. Thus, the two parameters were selected as the main inputs in the current study. The thickness of the workpiece was kept constant at 5 mm in all experiments to maintain a consistent thermal environment and ensure accurate interpretation of machining performance.

All experiments were conducted with deionized water as the dielectric medium, which ensures constant discharge conditions, effective flushing of debris, and efficient cooling of the spark gap. The electrode was a zinc-coated brass wire with a diameter of 300 μm. Zinc-coated wire was selected due to their better electrical conductivity, greater stability of sparking, and better flushing properties relative to standard brass wires. These features reduce wire breakage and enhance machining repeatability, providing consistent discharge behavior and experimental conditions.

### Measurement procedures

The processes and criteria applied to measurements in the present study are described to enhance the transparency and reproducibility of the experimental analysis. Table 1 displays the process parameters and their levels, and all other WEDM parameters, including discharge current, pulse-off time, wire feed (wire electrode run-off rate), dielectric flushing pressure, and gap voltage, were held constant in order to isolate the effects of the selected process parameters. The pulse-on time and servo voltage levels were selected based on preliminary machining trials and literature standards, and five levels were chosen to provide a broad operating range. This was done to ensure that sufficient experimental runs were conducted to adequately assess their effect on machining performance, particularly on surface integrity, material removal rate, and the phase transformation behavior of the TiNiCo alloy. The general workflow of the experiment and machining process is outlined in Fig. 2.

The average surface roughness (Ra) of machined surfaces was measured using a contact profilometer in accordance with ISO 4287, which provides the parameters and procedures for measuring surface texture on a standard basis. The surface roughness metric used in this study is the arithmetic mean roughness (Ra). The mass-loss method was used to determine the MRR, in accordance with the conventional machining evaluation practice outlined in ISO 3685, and the results were reported in mm^3^/min. The specimens were weighed before and after machining using a high-precision digital balance, and the mass difference was measured. The change in volume of the removed material was computed as the measured mass loss divided by the density of the TiNiCo alloy, and the MRR was calculated as the removed volume divided by the machining time. The results of the calculations are the MRR values in mm^3^/min. Table 2 presents the measured MRR and SR values across all experimental conditions.

**Table 1:** Input process parameters and their levels.

Parameters	Levels				
	1	2	3	4	5
Pulse on time (µs)	105	110	115	120	125
Servo voltage (V)	20	30	40	50	60
Pulse off time (µs)	42				
Wire run-off speed (m/min)	4				
Servo feed (µm/min)	2180				

**Table 2:** L_25_ Orthogonal array.

Run order	Pulse on time (µs)	Servo voltage (V)	SR (µm)	MRR (mm^3^/min)
1	105	20	1.51	3.71
2	105	30	1.46	3.12
3	105	40	1.33	2.28
4	105	50	1.19	1.89
5	105	60	1.11	1.50
6	110	20	2.29	4.42
7	110	30	2.20	3.19
8	110	40	2.03	2.21
9	110	50	1.76	2.19
10	110	60	1.67	1.84
11	115	20	3.80	2.72
12	115	30	2.26	5.01
13	115	40	2.25	3.71
14	115	50	2.41	2.86
15	115	60	2.15	2.51
16	120	20	3.85	5.32
17	120	30	2.57	6.31
18	120	40	3.43	5.58
19	120	50	2.35	3.58
20	120	60	2.32	2.81
21	125	20	5.01	8.43
22	125	30	3.20	8.65
23	125	40	3.07	8.06
24	125	50	2.41	5.20
25	125	60	2.39	3.38

**Fig. 3:**
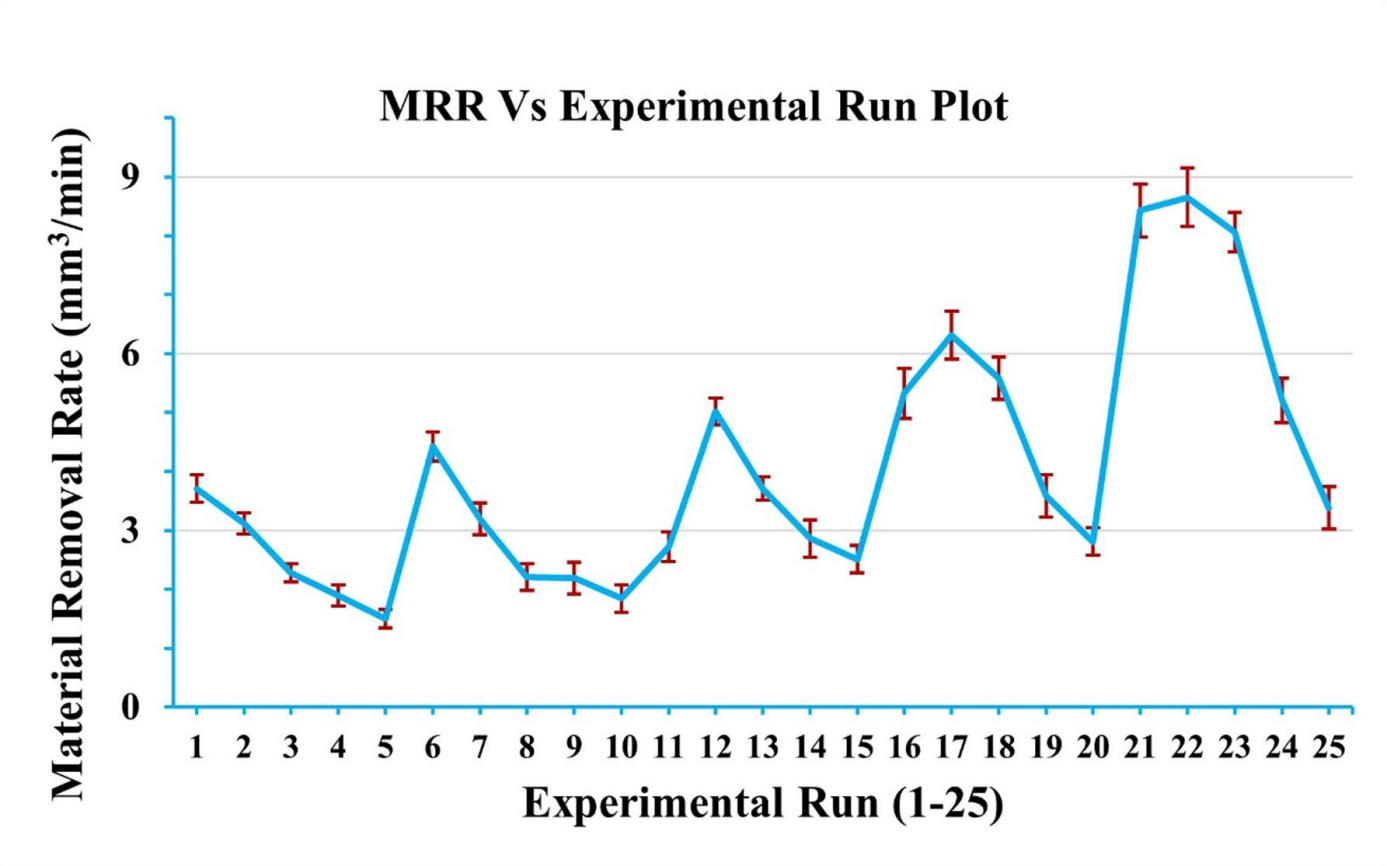
Effect of each run on the Material Removal rate.

**Fig. 4:**
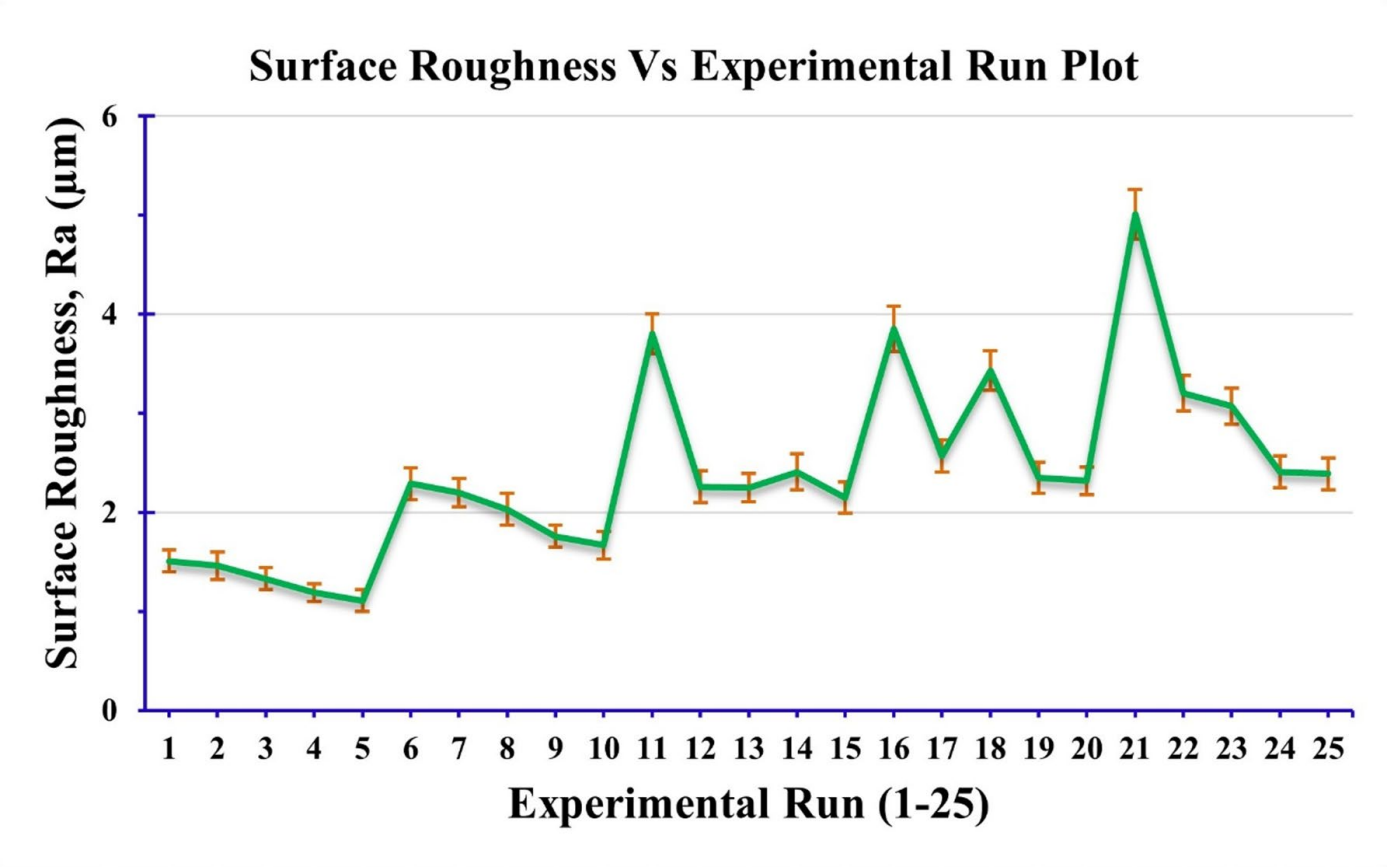
Effect of each run on the Surface roughness.

Three experimental runs were conducted to ensure reliable results. Analysis was performed using average values, and the variability of the measured responses was determined using the standard deviation. According to these repetitions, the values of the standard deviation of the MRR and the SR were calculated, and they are presented in Figs. 3 and 4, respectively.

## Result and discussion

### Effect of process parameters on machining Responses

Run order 21 had the highest MRR and SR, with 8.43 mm^3^/min MRR and 5.01 μm average roughness, as illustrated in Figs. 3 and 4. These results are associated with machining parameters applied in this run: 125 µs pulse-on time (Ton) and servo voltage (SV) 20 V. This parameter combination significantly contributed to the intensity and stability of discharge during the WEDM process and, in turn, has an impact on the machining performance and surface integrity.

The pulse-on time is one of the most important parameters in WEDM, governing discharge energy. As the pulse-on time increases, the duration of the electrical spark becomes longer. This consequently increased the energy output per pulse, improving melting and vaporization at the spark interface. This translates to an increased material removal rate. In the present work, the highest MRR of 8.43 mm^3^/min was achieved with a maximum pulse-on time of 125 µs. The discharge time is long, resulting in greater thermal energy transfer to the workpiece and deeper, wider craters on the machined surface of Ti₅₀Ni₄₉Co₁ alloy.

Increasing pulse-on time improves MRR, but surface quality is compromised. A long discharge period results in localized heating, leading to overheating of the workpiece surface. The pulse-off time and flushing pressure are constant, so that much of the molten material does not completely leave the spark gap. Instead, as the surface solidifies, some fraction of the molten debris, as it cools, recasts layers and microglobules that are typically visible in SEM images of WEDM-machined surfaces. This behavior explain the higher SR in Run order 21 (Ra = 5.01 μm).

Flushing efficiency also influences this behavior. Long pulse-on times and a higher rate of molten material are obtained. However, if the dielectric fluid fails to remove this entire molten material in the machining area, random sparking and secondary discharges may occur. These secondary discharges create uneven craters and localized remelting zones, further aggravating the surface finish. Therefore, MRR enhancement often leads to a decrease in surface smoothness.

In addition to pulse-on time, servo voltage is also an important factor in machining performance and surface quality. The servo voltage largely controls the distance between the wire electrode and the workpiece. As the servo voltage increases, the spark gap widens, reducing discharge energy and minimizing material erosion. Thus, when the servo voltage is high, the surfaces will be smoother but with low MRR. However, in the present investigation (Run order 21), both the long pulse-on time and the high MRR were the most prominent machining features, even though the intermediate servo voltage was 20 V, resulting in high MRR and increased surface roughness.

These results demonstrate that productivity in terms of MRR and surface integrity in terms of (Ra) in WEDM are in trade-off. When discharge energy parameters, i.e., pulse-on time, are increased, productivity increases at the cost of surface finish as the thermal action heightens. Conversely, it is possible to control the voltage across the spark gap or to minimize the discharge energy to improve surface finish but decrease cutting performance during WEDM. Therefore, this requires selecting the most appropriate parameters based on the functionality of the machined component.

The identified trends align well with the literature. Manjaiah et al.^25^ studied the WEDM of TiNi-based shape memory alloys and demonstrated that pulse-on time has a positive impact on MRR and a negative impact on surface quality due to over-melting and resolidification. They also discovered that the discharge energy was inversely related to the thickness of the recast layers and the surface roughness.

Further, the SMAs made of TiNi exhibit thermoelastic martensitic transformations that dictate their machining behavior. WEDM involves rapid heating and cooling rates during material removal process, which can cause residual stresses and phase transformations, altering the surface morphology and microstructure. All these effects are further augmented at high discharge energies and suboptimal flushing conditions, which account for the augmented surface roughness in Run order 21.

In practice, this means the pulse-on time may be long to enhance machining efficiency, but it should be optimized to avoid excessive surface damage. Lower pulse-on times with controlled servo voltage would be preferable to achieve smoother surfaces and slower recast formation (particularly in high-precision applications), e.g., in biomedical or aerospace components of TiNi SMAs. Alternatively, the pulse-on times could be increased in order to maximize productivity in rough-cutting processes.

In general, Figs. 3 and 4 indicate that Run Order 21 had the largest energy input, which occurred at a long pulse-on time and a low servo voltage. The experimental MRR of 8.43 mm³/min and Ra of 5.01 μm clearly demonstrate that discharge energy is a significant factor influencing machining performance as well as surface features. The results confirm the two-sided nature of WEDM discharge parameters, such that higher energy leads to greater productivity but lower surface quality, and vice versa, which emphasizes the necessity of optimizing these parameters.

### Confocal microscopy

**Fig. 5:**
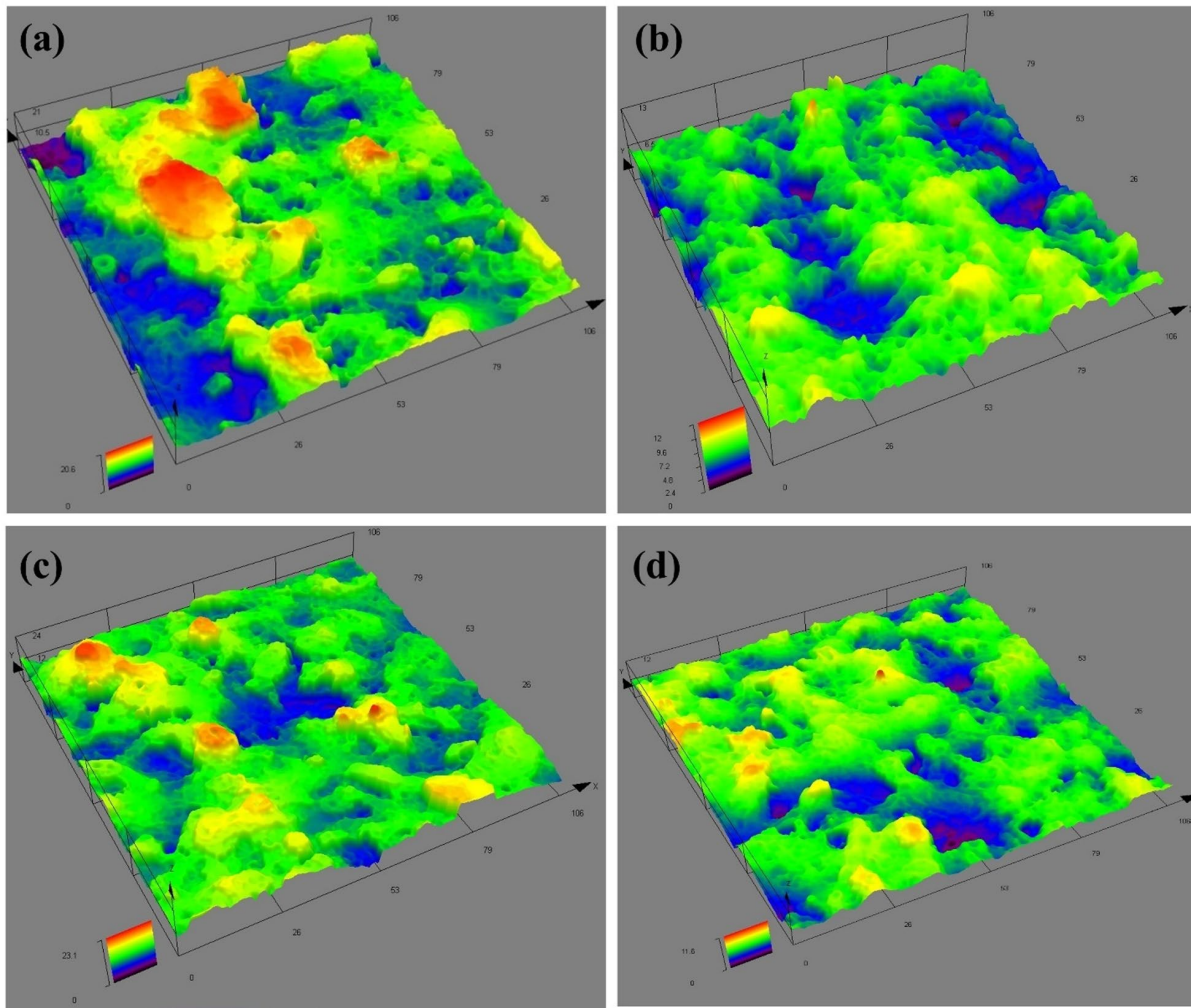
Confocal microscopy images showing the machined surface topography of the Ti₅₀Ni₄₉Co₁ alloy under different WEDM conditions: (**a**) low servo voltage, (**b**) high servo voltage, (**c**) high pulse-on time, and (**d**) low pulse-on time.

Confocal microscopy was used to examine the machined surface topography under different machining conditions: low servo voltage in Fig. 5(a), high servo voltage in Fig. 5(b), high pulse-on time in Fig. 5(c), and low pulse-on time in Fig. 5(d). The analysis focused on the peak-to-valley height corresponding to the surface roughness parameter Rz, which represents the vertical distance between the highest peak and the lowest valley in the measured profile. The three-dimensional pseudo-color maps were obtained at 100X magnification over a 106 μm × 106 μm scan area, illustrating the significant morphological variations produced by the thermo-electrical erosion process during WEDM.

Under low servo voltage shown in Fig. 5(a), the peak-to-valley height (Rz) reached 20.6 μm, indicating unstable and energetic sparking conditions that generated deeper craters and increased recast layer deposition. The high servo voltage condition in Fig. 5(b) resulted in a 12 μm Rz value, attributed to the stability of the sparks and the lower discharge intensity, which prevented excessive melting and re-solidification. The effect of discharge duration can be observed at maximum pulse-on time, as shown in Fig. 5(c), where the largest Rz height of 23.1 μm is observed, due to greater energy input and faster crater formation. Meanwhile, the low pulse-on time condition, as shown in Fig. 5(d), results in a much lower Rz value of 11.6 μm, indicating less thermal loading and melt ejection areas.

On the whole, the observations indicate that there is a close relationship among discharge energy, plasma channel behavior, and recast layer morphology, which are mainly determined by pulse-on time and servo voltage in deionized water.

### XRD analysis

**Fig. 6:**
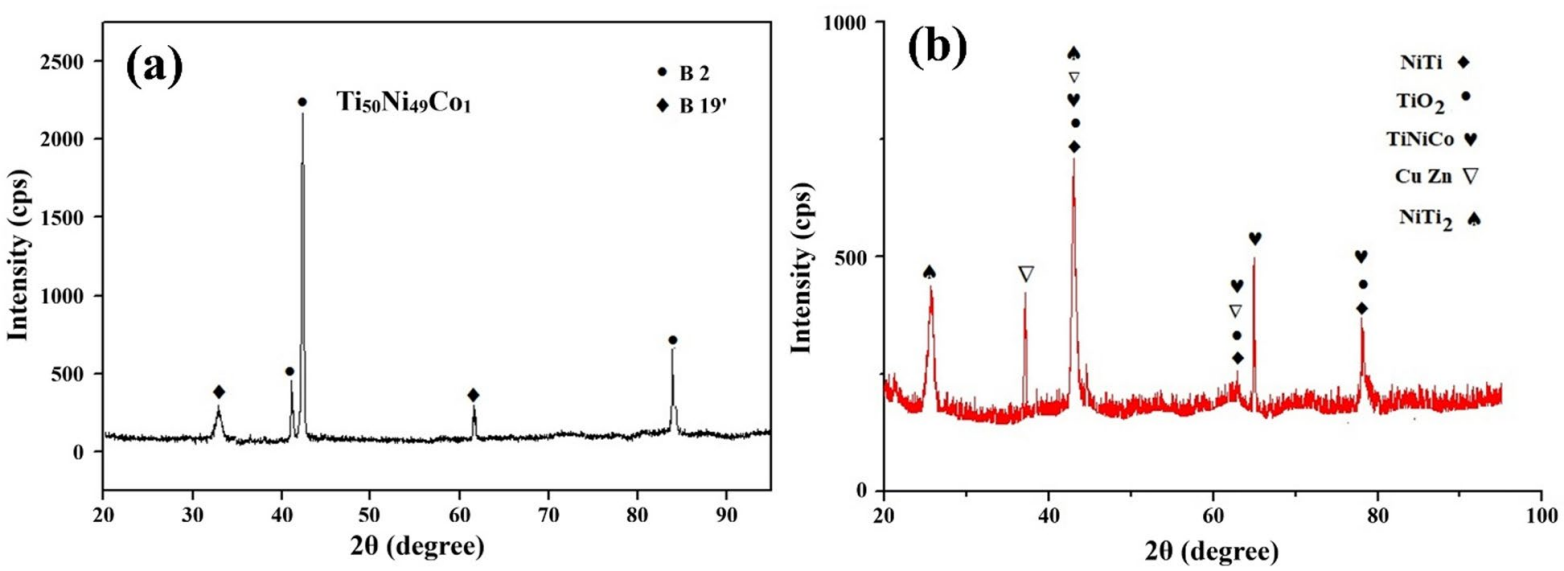
XRD patterns of (**a**) as-cast Ti₅₀Ni₄₉Co₁ alloy and (**b**) the machined surface.

Figure 6(a–b) shows the X-ray diffraction (XRD) patterns of the as-cast Ti₅₀Ni₄₉Co₁ alloy and the WEDM-machined surface, respectively. The as-received Ti₅₀Ni₄₉Co₁ substrate in Fig. 6(a) exhibits the dominant B_2_ austenite phase along with minor B19′ martensite peaks. In contrast, the machined surface in Fig. 6(b) reveals additional phases such as TiO₂, NiTi₂, and CuZn, which are attributed to oxidation and possible material transfer from the brass wire electrode during the discharge process. Considering the typical XRD penetration depth of approximately 1–10 μm, the detected phases correspond to the recast and thermally affected surface layer generated during machining.

The XRD diffractogram shown in Fig. 6 reveals several crystal phases, i.e., NiTi (B_2_), NiTi_2_, TiO_2_, TiNiCo intermetallics, and minor CuZn compounds, resulting from compositional changes caused by machining and electrode contact. The sharp NiTi peaks at around 42º, 61º, and 78º provide evidence that the B_2_ austenitic parent phase remains dominant after machining, but other secondary phases are also observed in the recast layer.

Selective evaporation of Ni at high discharge energy is the main reason, as Ni has a lower boiling point than Ti, which leads to the formation of NiTi_2_. Such selective Ni loss creates Ti-enriched areas that facilitate the development of Ti-enriched intermetallic compounds during the rapid solidification. At the same time, TiO_2_ is formed by the oxidation due to oxygen diffusion in the high-temperature plasma channel, producing a stable oxide layer on the recast surface. Such changes alter the surface stoichiometry and reduce the effective fraction of transformable NiTi matrix, consistent with the lower transformation enthalpy observed in the DSC study.

To further observe micro-globules, discontinuous molten craters, and resolidified debris, as common microstructures of the WEDM-processed surface, confocal 3D microscopy is used. The brittle intermetallic layers of NiTi_2_ and the oxide layers also result in the hardness and roughness of the surface of the machined product, whereas the presence of the CuZn phase indicates the inclusion of electrode debris of the brass wire in its machining.

These findings agree with the DSC results, which show small changes in the martensitic start (M_s_) and finish (M_f_) temperatures, along with a decrease in transformation enthalpy. These are caused by microstructural transformations, such as the formation of Ni-deficient NiTi_2_ phases and TiO_2_ layers, which, to some extent, prevent the reversible martensitic transformation. However, it is, nevertheless, possible that the fundamental shape-memory functionality of the alloy is maintained, as the retained B_2_ and TiNiCo phases indicate that the fundamental shape-memory functionality is preserved. The broadened peaks of transformation are linked to residual stresses and lattice distortions induced by rapid thermal cycling and solidification during the WEDM process. All in all, the results of combined XRD, DSC, and confocal microscopy indicate that WEDM can alter the surface microstructure and phase redistribution of TiNiCo SMA by oxidizing and redistributing its phases, forming recast layers, but the alloy’s main thermoelastic transformation capability is preserved.

### DSC analysis

**Fig. 7:**
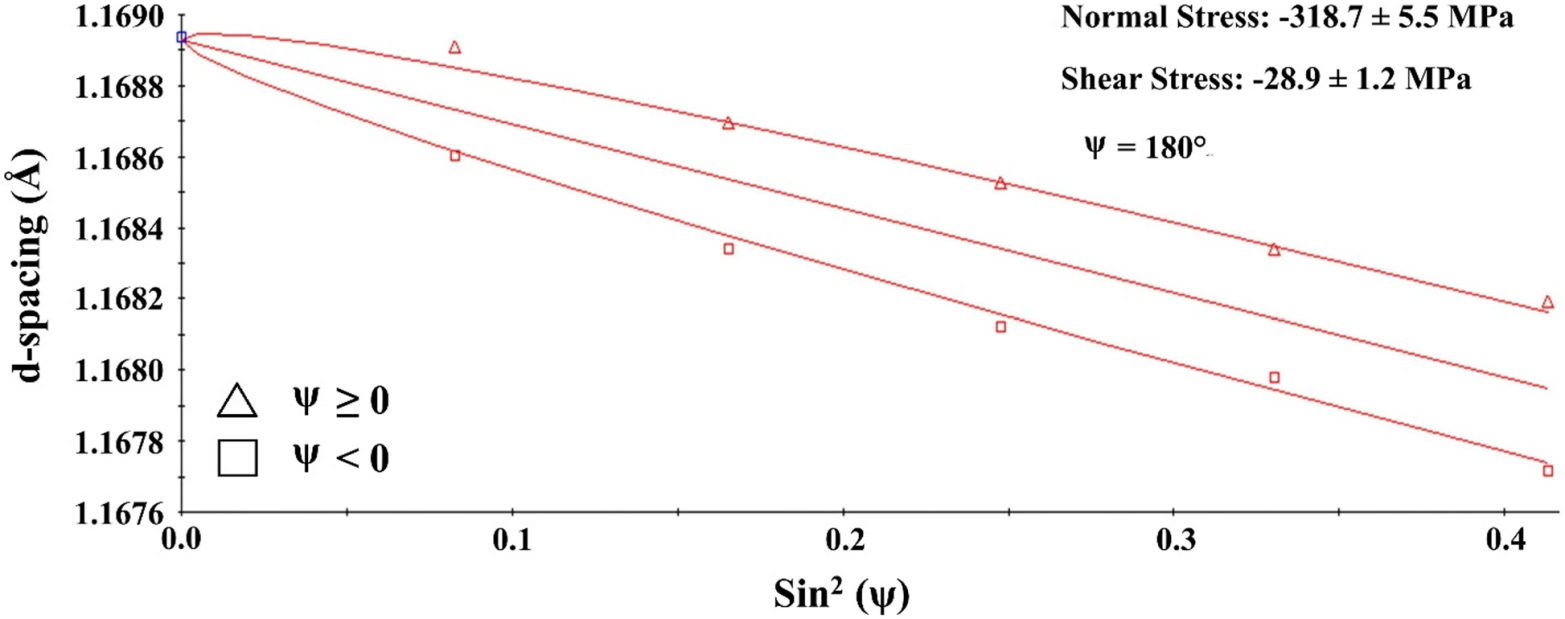
DSC analysis of the machined surface of TiNiCo shape memory alloys.

The DSC response of the electro-discharge machined TiNiCo alloy (Fig. 7) shows that the transformation behavior of the alloy is not the same as that of the base material. The presence of distinct transformation peaks during both heating and cooling cycles confirms that the martensitic transformation remains active after WEDM processing. However, noticeable changes in peak shape and intensity suggest that the transformation characteristics have been modified. These observations indicate that the martensitic transformation was partially suppressed in the machined layer, likely due to microstructural alterations and residual stresses induced during the WEDM process.

The selective evaporation of Ni in cases of intense energy discharge can explain this change in the transformation properties because Ni has a lower boiling point than Ti. The loss of Ni in the local molten zone leads to Ti enrichment in the near surface. This Ti-rich alloy favors the development of a Ti-rich intermetallic phase, such as NiTi₂, during rapid solidification. Besides, local melting of elements in the molten pool owing to electrical discharges during machining results in the redistribution of the elements. The formation of a TiO₂ layer on the recast surface may also affect the stability of the NiTi matrix and inhibit the movement of martensitic interfaces. These microstructural changes contribute to the change in martensitic transformation behavior that occurs. In addition, compressive normal stress (− 318.7 ± 5.5 MPa) and small shear stress (− 28.9 ± 1.2 MPa) can also be observed in the residual stress analysis using the Sin²ψ approach (Fig. 7), which may additionally influence transformation temperatures and kinetics.

Surface characteristics of the rapid melting and solidification during spark erosion are observed as microcraters, re-solidified droplets, debris islands, and heat-affected regions. It is found that these microstructural abnormalities cause local strain gradients and residual thermal stresses, which lead to changes in phase transformation kinetics and hysteresis, as shown in the DSC curves. The recast layer acts as a diffusion-altered barrier that impedes interface mobility and slows the transformation process. Also, with machining parameters, e.g., increased pulse-on time and servo voltage, the MRR increases, but the surface roughness and heating effects, which make the recast layer thicker and surface disruptions more pronounced, also increase. Consequently, transformation peaks become broader, transformation enthalpy is decreased, and the martensitic transformation has been clearly shown to depend on process energy input, surface integrity, and microstructural quality.

Overall, the results of the combined DSC, XRD, and confocal microscopy reveal that WEDM alters the martensitic transformation of TiNiCo alloys by altering the surface chemistry, leading to the appearance of microstructural anomalies and the development of residual stresses. Although the basic shape-memory capability has been maintained, the martensitic transformation at the machined surface is somewhat inhibited, and its performance largely depends on the machining environment and surface integrity.

## Conclusion

XRD, DSC, and 3D surface profiling analyses were systematically applied to TiNiCo shape memory alloy processed by WEDM. The results indicated that thermal-electrical interactions during machining had a significant impact on the morphology of the alloy surface and its functional response. At high discharge energy, particularly at a pulse-on time of 125 µs and a servo voltage of 20 V (Run order 21), the high level of surface melting and rapid re-solidification resulted in a peak MRR of 8.43 mm³/min and a maximum SR of 5.01 μm. The depth of craters, the recast layers, and re-solidified debris were greater in the 3D surface projections at high energy input. XRD indicated the secondary phases, including NiTi_2_, TiO_2_, and CuZn compounds as oxidation and transfer of the electrode material. The DSC results showed broadened peaks, and variable transformation temperatures, consistent with the conclusion that the martensitic transformation was partially suppressed in the machined layer. Functional stability and surface integrity were seriously affected, but higher pulse-on time enhanced productivity. Therefore, the optimization of the servo voltage and Ton is needed to achieve stable performance and accuracy in actuator, biomedical, and smart engineering systems. The compositional change and oxide formation partially suppress the martensitic transformation in the recast layer, and the bulk retains some of the required shape-memory functionality with different transformation temperatures.

The most important Experimental results are as follows:

- The highest pulse-on time (Ton) of 125 µs and servo voltage (SV) of 20 V were used as the MRR was 8.43 mm^3^/min, which is a good indication of high discharge energy input.
- The highest surface roughness (Ra) of 5.01 μm was obtained under the same machining condition, indicating that there was profound wear on the surface caused by high-intensity spark erosion.
- 3D surface profiling showed that there was a deeper crater formation and a thicker recast layer, which further confirms that much melting and a quick re-solidification took place during machining.
- Also, XRD analysis revealed the presence of secondary phases like NiTi_2_, TiO_2_, and CuZn, which were formed as a result of selective evaporation, oxidation, and transfer of electrode material.
- DSC analysis showed reduced transformation enthalpy and slight shifts in transformation temperatures, indicating partial suppression of martensitic transformation at the machined surface.

## Data Availability

The data used to support the findings of this study are included within the article.
